# Wildlife use and the role of taboos in the conservation of wildlife around the Nkwende Hills Forest Reserve; South-west Cameroon

**DOI:** 10.1186/1746-4269-11-2

**Published:** 2015-01-07

**Authors:** Kadiri Serge Bobo, Fodjou Florence Mariam Aghomo, Bonito Chia Ntumwel

**Affiliations:** Department of Forestry, Faculty of Agronomy and Agricultural Sciences, University of Dschang, P.O. Box: 222, Dschang, Cameroon; School for the Training of Wildlife Specialists Garoua, Ministry of Forestry and Wildlife, P.O. Box: 271, Garoua, Cameroon

**Keywords:** Cameroon, Culture, Ngunnchang and Obang communities, Nkwende hill forest reserve, Taboos, Traditional ecological knowledge, Wildlife

## Abstract

**Background:**

Cameroon is known as Africa in miniature because of its multitude of ecosystems and associated biodiversity, cultures and traditions. The country also harbors very ancient human populations whose relationship with nature is very intimate and where animals play important roles for their livelihood. Located in the South-west region of Cameroon, the Nkwende Hills Forest Reserve (NHFR) represents an important wildlife conservation site because of its strategic position at the periphery of Korup National Park (KNP). The periphery of NHFR is inhabited by several ethnic groups amongst which are the Obang and Ngunnchang clans who share particular relationships with wildlife. The present paper studies these relationships and contributes to the growing trend of scientific ethnozoological studies across Africa.

**Method:**

From August to December 2011, a questionnaire survey was addressed to 126 randomly chosen household respondents (HRs) in seven villages at the Northwest periphery of NHFR. In households, preference was given to parents, and to the eldest child in case the parents were absent. Questions related to the uses and local taboos on wildlife species were asked to HRs.

**Results:**

Both communities have accumulated knowledge on the use of 51 wildlife species of which 50.9% represent mammals, 21.6% birds, 15.7% reptiles, 7.8% fish and 3.9% invertebrates. Four main use categories of wildlife by both communities were identified, namely (1) Food, medicine and sales values (41.2%), (2) Ethnomusical animals and parts used as trophy (29.2%), (3) Decoration and jewelry making values (21.9%) and (4) Magico-religious and multipurpose values (7.8%). Regarding local taboos, species specific taboos (generation totems and acquired totems), habitat taboos (sacred forests), method and segment taboos still persist but are rarely respected among the youth mainly because of the scarcity of wildlife (65.3% of HRs).

**Conclusion:**

Like other communities living around forest areas, the studied communities use wildlife in their culture and tradition. Wildlife is not only used for consumption, but also for traditional medicines, craft materials and spiritual purposes. But, threats to wildlife and their traditional uses are real and acculturation seems to be the main driver. High priority should be given to the reconciling conservation of species with high values for local communities and human needs.

## Background

Tropical evergreen forests are the most species-rich ecological ecosystems and are highly endangered all over the world [1]. According to [2–5], Cameroon forests are primordial for the conservation of African biodiversity, but continue to face alarming threats. Some of these threats are closely related to people’s dependency on Non Timber Forest Products for their livelihoods [6–8]. Taking into account the importance of biodiversity in our communities today [9], integrated conservation measures for sustainable management seems to be the best solution [10, 11]. The adoption of such measures requires knowledge from local populations who are the principal indicators of the changes observed in their area [12]. Ethnobiology helps adapt a link between natural resources and local populations and requires two basic components i.e. the knowledge on biodiversity and its uses (mammals, birds, reptiles and amphibians) and a comprehension of the culture [13, 14]. It has been demonstrated that these uses are generally guided by laws and restrictions important for conservation [15, 16]. Several studies have shown how these uses, cultures and traditions lead to wildlife conservation [13, 17, 18]. Also, the culture and tradition regulate the use of certain species especially those of nutritional and medicinal importance [19–22]. Ethnozoological studies can be a valuable asset to increase our understanding of the economic, cultural, social, and traditional roles played by animals and reconcile human and conservation needs. In this context, they have a central role in conservation and management [23]. Increased consideration for traditional uses of wildlife will help solve important conservation problems related to human-wildlife conflicts [24]. Therefore, to secure a future for animal populations, conservationists must understand not only the ecological, but also the cultural and economic interactions that link ecological and social systems [25]. Furthermore, there is a great need for ethnobiological studies in Cameroon given the increasing relevance of this science across Africa. The present paper documents traditional uses and cultural values of wildlife for local populations, and the contribution of taboos to wildlife conservation around the NHFR.

## Methods

### Study area

**Figure 1:**
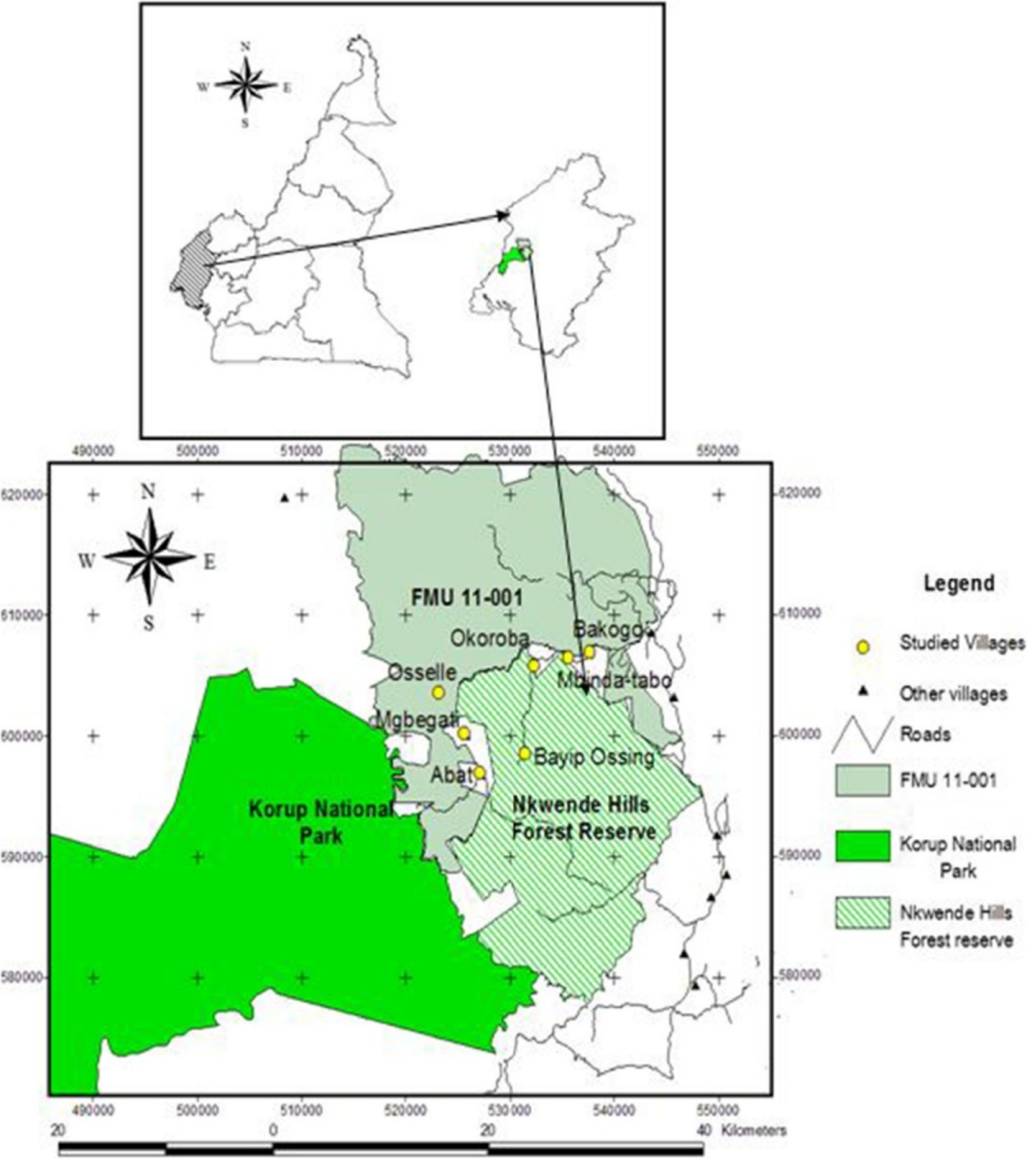
**The study area in the map of Cameroon, showing the studied villages.**

The present study was carried out in seven villages (Abat, Mgbegati, Bayib-ossing, Osselle, Okoroba, Mbinda-Tabo and Bakogo) situated in the support zone of KNP, specifically between the Northwestern part of NHFR and Northeastern part of KNP, in the Southwest region of Cameroon (Figure 1). The name “Nkwende” in Ejagham tribe, meaning “a water source for animals”, was given to those hills in 1960. The Nkwende hills lie to the Northwest of Nguti town and to the West of Nguti-Mamfe road. The highest peak is about 737 m asl around Bayib-Ossing village [26]. Part of NHFR extends to Okoroba village recognisable by rock faces commonly called by the locals “chimpanzee stone”, alleged to be a refuge for chimpanzees. The forest at present is a secondary forest in the surrounding villages due to logging and agricultural activities. Intact primary forests still exist in higher altitudes where no suitable timber trees were found or where the sloppy nature of the hills made forest exploitation impossible [26].

Due to its proximity to KNP, the study area shares similar climatic, fauna and flora characteristics. The rainfall is about 5000 mm per year and the mean annual temperature is about 25°C [27]. The large mammal fauna of KNP consists of 33 families with 161 species. KNP contains one quarter of all Africa’s primate species and represents an important site for primate conservation. It contains species with restricted distribution including a number of endemics such as the giant otter shrew *Potamogale velox*, Calabar angwantibo *Arctocebus calabarensis*, drill *Mandrillus leucophaeus*, and Preuss’s red colobus *Procolobus preussi*. Concerning small mammals, Korup contains as many as 55 species of bats and 47 species of rodents. Here, the presence of three shrews (*Crocidura crenata, C. grandiceps and C. lamottei)* were recorded for the first time in Cameroon and a new species was discovered *Sylvisorex pluvialis* [28]. In ornithological terms, KNP is reputedly the most diverse lowland site in Africa with a total of 420 bird species recorded so far in 53 families [29–31]. Four species found in the area are considered to be ‘rare’ including Green-breasted bush-shrike *Malaconotus gladiator*, White-throated mountain-babbler *Lioptilis gilberti,* Red-headed rockfowl *Picathartes oreas* and Yellow-footed honeyguide *Melignomon eisentrauti.* The African grey parrot *Psittacus erithacus* is heavily hunted for export trade. In addition, Korup contains 82 reptile and 92 amphibian species, a number of them being endemic to the area. They include three caecilian species, 89 species of frog and toad, two species of tortoises, two species of aquatic turtles, 15 species of lizards, five species of chameleons, three species of crocodiles and 55 species of snakes. Amphibians listed as endangered or vulnerable include *Amietophrynus superciliaris* and *Nectophryne afra* [32, 33]. Finally, regarding fish, rivers draining Korup and Nkwende are not uniform in their taxonomic composition and diversity. Peculiar to this zone are colonies of sting-rays, typically marine snappers *Lutjanus sp*.; and a jack *Trachinotus goreensis*, all living over 300 km from the sea. About 130 species of fish are known from the area [34].

Socio-economically speaking, the social organisation of the village around the NHFR is made of a chief, regent chief (traditional) and other community-based structures. Although important decisions are usually taken in consultation with the traditional council and regent chief, the juju society locally called “Ekpe”, remains the most important social institution and governing body in all villages of the support zone of KNP [35–38]. Community based institutions for the management of natural resources, such as Forest Management Committee (FMC) and other village development associations, exist in the study area. The inhabitants are the Lower Oban people, precisely the Ngunnchang and Obang clans. The common communication language is Pidgin English and the traditional spoken language is the Ejagham.

### Socioeconomic profile of Household Respondents (HRs)

#### Distribution of HRs per gender and age

**Figure 2:**
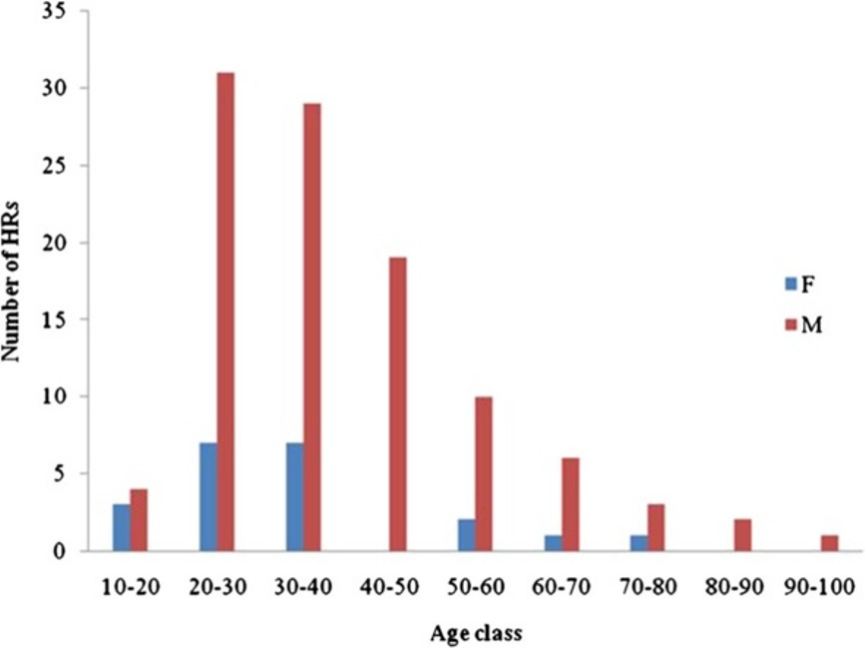
**Distribution of HRs per gender and age.**

HRs were dominantly males of the age group 20 to 40 years old (Figure 2). This represents the active age group. Females were less represented because during interviews, preference was given to males who were considered to be the head of the household, originating from the village, principal hunters and having more knowledge concerning culture and traditions in relation to wildlife.

#### Distribution of HR by level of education

**Figure 3:**
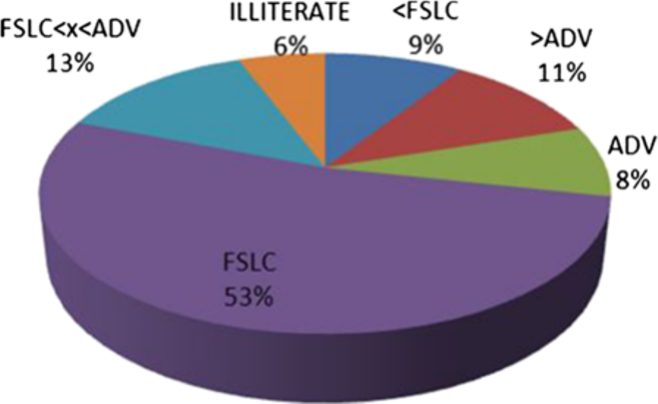
**Distribution of HRs by level of education.** Notes: FSLC refers to HRs who are holders of the First School Leaving Certificate; ADV refers to HRs who are holders of a General Certificate of Education - Advanced Level and ILLITERATES refers to HRs who have not been to school; FSLC < X < ADV refers to HRs who attended secondary education but did not obtain the ADV; <FSLC refers to HRs who attended primary education but did not obtain a FSLC; >ADV refers to HRs who attended higher education after the ADV.

About 94% of HRs went to school. Among these, 53% have attained the First School Living Certificate (FSLC) at the end of their primary education. This high proportion of HRs with the FSLC is explained by the absence of secondary schools in the area [39, 40]. About 11% of HRs have gone to university, i.e. having more than the General Certificate of Education - Advance Level (>ADV), and 6% were “Illiterates” i.e. have not gone to school at all (Figure 3).

#### Religion of HRs

**Figure 4:**
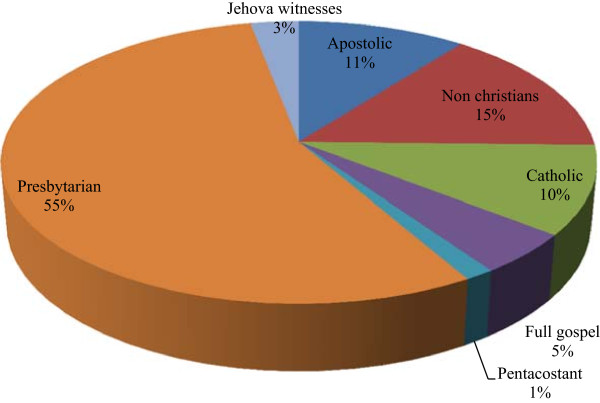
**Distribution of HRs per religion.**

About 85% of HRs are Christians. Among these, 55% are Presbyterian Christians (Figure 4). These religions greatly influence the uses of wildlife in the culture and tradition. For example, Jehovah witnesses do not use wildlife because it is prohibited by their religion. On the other hand, non Christians continue to practice traditional rights and respect their tradition. Irrespective of the religious affiliation, HRs use wildlife for traditional purposes in the study area.

#### Economic activities

**Figure 5:**
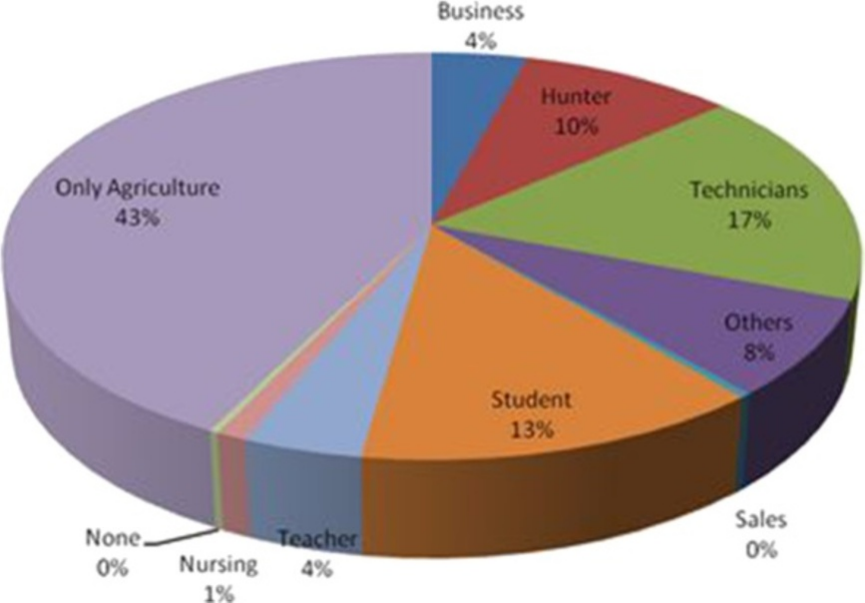
**Distribution of HRs per economic activity.**

In Cameroon, agriculture represents the primary production sector and it is the main source of employment. It contributes significantly to the Gross Domestic Product of the nation [41]. In the study area, 43% of HRs practiced only agriculture. Main agricultural products in the study area are cocoa *Theobroma cacao*, plantain *Musa spp*.; oil palm *Elaeis guineensis*, cocoyam *Colocasia esculentum,* cassava *Mahinot spp*.; groundnut *Arachis hypogea* and maize *Zea mays*. Livestock production is poor in the study area and few goats, chickens, pigs and cattle constitute the main livestock products (as in [37]). People practicing other activities in association with agriculture (“Others”) represent 8% of HRs. About 13% and 10% of HRs are students and hunters respectively (Figure 5).

### Data collection

A questionnaire survey was carried out in 126 randomly chosen households from the seven studied villages. Households were chosen by simple random sampling based on the size of the village. It consisted in counting the number of households in each of the seven studied villages, producing a sketch map with the positions and numbers of each household, then numbering them on papers, and then proceeding in a draw without replacement. For villages with households between 0 and 20, a 100% sampling was performed, between 21 and 50, a 50% sampling was performed, and greater than 50, a 25% sampling was performed. Globally, the sampled households in the studied villages ranged between 25% and 57.1%. HRs were any of the parents or eldest child met at home. Within a household, preference was given to the family head who was considered to be the eldest, the principal hunter, originating from the village, and then supposed to know more concerning the culture and traditions of the village in relation to wildlife. In case of his absence, preference was given to the mother. If both parents were absent, the eldest child was interviewed. In each household, questions were asked to assess their awareness about wildlife used in their culture and as local taboos. Furthermore, self observation and discussions with villagers of all age and sex during meetings, community works and football occasions helped for cross checking. Wildlife species were identified thanks to local names given by HRs, and with reference to the field guide of African mammals [42].

Prior to the realisation of the study, the Chief of the village and the head of the family were consulted and their verbal approval was obtained before any interview. They are aware of the possibility of writing any publication from the data collected and wish to receive a copy of the publication when ready.

## Results and discussions

### Traditional uses of wildlife

Like in other areas of Central Africa, the primary use of wildlife in the study area is for consumption. This is because many people depend on bushmeat as a means to survive during time of hardship (e.g. unemployment and crop failure), or to gain additional income for special needs (e.g. school fees, festivals and funerals) [43]. This ‘safety net’ is often more important for the more vulnerable members of the community [15]. Traditional hunting of wildlife in Cameroon for subsistence purposes is not prohibited, though the wildlife law addresses restrictions on the class of species to be harvested, places of harvest, harvest methods, type of weapons and quantity/final use of product [44]. As concerns consumptive uses of wildlife, the most consumed mammal species are Brush-tailed porcupine *Atherurus africanus,* Blue duiker *Cephalophus monticola,* Ogilby’s duiker *Cephalophus ogilbyi,* Pangolin *Uranamis tetradactyla* and monkeys *Cercopithecus spp.*; (see also [40, 45–47]). Consumed reptiles are African rock python *Python sebae* and Rhinoceros horned viper *Bitis nasicornis.* The birds consumed are generally large in size such as hornbills *Tockus spp.;* African grey parrot *Psittacus erithacus* and Black guineafowl *Ageslates niger.* As concerns amphibians, toads *Bufo spp.*; is the most consumed. Lastly, the most consumed fish species are Tilapia *Tilapia spp*.;, *Sarotherodon sp.*; and mudfish *Heterobranchus sp*;.

Apart from their consumptive uses, 99.7% of HRs in the study area recognised the effective use of wildlife in their culture and traditions. It was found that 26 mammal species (Table 1, Figures 6, 7, 8, 9, 10, 11, 12, 13, 14, 15, 16), 11 bird species (Table 2, Figures 17, 18, 19), eight reptile species (Table 3, Figures 20, 21, 22), four fish species (Table 4) and two invertebrates (Table 5), representing respectively 50.9%, 21.6%, 15.7%, 7.8% and 3.9% of all animal groups, are used in various ways for cultural and traditional purposes.

**Table 1:** **Mammals parts used and reasons**

Animal	Part used	Use(s) and use method(s)	Reason(s)
Peter’s duiker *Cephalophus callipygus*	Teeth^3^	- Design necklaces	For prestige
	Skin^2,3^	- Making of drums	Durable, produces the desired sound, easily malleable and it is an inherited practice from the elders. Also, the skin is not good for consumption.
		- Decorating houses of Ekpe society members during liberation ceremonies	Traditional inherited practice
	Meat^1^	- Consumed by pregnant women for blood regeneration	For better development of the foetus
African forest buffalo *Syncerus caffer*	Horn^1,2,3^ (Figure 6)	- Musical instrument	Announcing bad news
		- For drinking wine (traditional cup) (see also [48])	Has the shape of a cup
		- Sold to Nigerians	For money
		- Design necklaces	Traditional inherited practice
	Skull^3^	- Decoration	Traditional inherited practice
	Bones^1,4^	- Used by sorcerers	Not revealed
		- Treats goiter: grind the bones and mix with palm kernel oil (manyanga) and apply on goiter locally called “nkongho illness”	Medicinal
	Limbs^1^	- Treats abscesses: mix with oil palm and apply on abscess	Medicinal
	Skin^2,3^	- Making of drums	Resistant
		- Decoration	Shows hierarchy between members of the Ekpe society
	Meat^1^	- Consumed	Believed to treat goiter patients
Yellow back duiker *Cephalophus sylvicultor*	Skin^1^	- Treat skin inflammations: apply on swollen parts	Medicinal
Bay duiker *Cephalophus dorsalis*	Skin^2,3^	- Decorate the seat of the chief of Ekpe society	Denotes hierarchy
		- Making of drums	Resistant
Blue duiker *Cephalophus monticola*	Skull^1,3^	- Sold to Nigerians and used to design necklaces	For money
	Jaws^1^	- Treats tooth ache and intestinal worm problems: burn, grind and mix with leaves of *Aframomum melegueta* locally called “alakata pepper”	Medicinal
	Bones^1^	- Sold to Nigerians	For money
	Skin^2^	- Making of drums (Figure 7)	Elastic, easily malleable, has little or no fats, light, durable and resistant
		- Decorate the seat of the chief of Ekpe society	Denotes hierarchy
	Hoofs^1^	- For purging children	Renders the child strong and active
	Meat^1,4^	- For liberation ceremonies: cook with plantains *Musa sp*.; and/or cassava *Manihot esculenta* and given to ancestors	Considered as a dead animal and can link the living to the dead
		- Marriage ceremonies: cook with plantains	Traditional inherited practice
		- Consume during death and traditional dance ceremonies	Easily hunted
Ogilby’s duiker *Cephalophus ogilbyi*	Horn, tail limbs, Skull and hoofs^1,3^	- Sold to Nigerians	For money
		- Decoration	Traditional inherited practice
	Skull^1^	- Treats frontal headache: burn, grind and apply on the forehead	Medicinal
	Oil from the bones^1^	- Treats inflammations: apply on swollen parts	Medicinal
	Jaws^1^	- Treats toothache and intestinal worms: burn, grind and mix with *Aframomum melegueta* and consume	Medicinal
	Bones^1,2^	- Musical instrument: used together with the shell of tortoise	The bones are big
		- Sold to Nigerians	For money
	Skin^2^	- Making of drums	Produces a good and desired sound, durable, light and elastic. Has no fats and the skin is not good for consumption
	Meat^4^	- For liberation, marriage and death ceremonies to appease and communicate with ancestors: during such ceremonies, it is cooked with plantains. During incantations, part of the meal is poured on the ground (around graves, sacred sites) together with palm wine (*Raphia sp*.;), oil palm, water, tobacco, cola nuts (*Cola acuminate*) and coins	It is considered as the incarnation of dead people and can act as a mean of communication between the living and the spirits of deaths. It is also used because goats are scarce and very expensive
Red-capped mangabey *Cercocebus torquatus*	Skull^1^	- Sold to Nigerians	For money
	Head^1^	- Treats tuberculosis: burn, grind and mix with oil palm and consume	Medicinal
Hammered bat *Hypsignathus monstrosus*	Fur^1^	- Treats burns: plaster on the burned parts	Medicinal
Water chevrotain *Hyemoschus aquaticus*	Limbs^1^	- Sold to Nigerians and to tradi-practitioners	For money
	Meat^4^	- Most used during ceremonies: cook in meals	Traditional inherited practice
	Bones of limbs^1^	- Treats fractures: apply on fractures of the legs	Medicinal
	Skin^2^	- Making of drums	Resistant and produces the desired sound
	Veins of limbs^1^	- Treats paralyses: mix with roots of *Aframomum melegueta.* The paste produced is applied on cuts made on the body using the teeth of viper	Medicinal
	Meat^4^	- Liberation ceremonies: cooked with Ogilby’s duiker and other medicines. During incantations, part of the meal is poured on the ground around graves, sacred sites	Traditional inherited practice
Ellioti chimpanzee *Pan troglodytes*	Skull, bones and limbs^1^	- Sold to Nigerians	For money
		- Fortify men: grind and mix with *Aframomum melegueta,* and applied on cuts made on the body with a razor blade	Believed to be a strong animal
	Skull^3^	- Making of necklaces	For money
	Hands and limbs^4^	- Consume mainly by chiefs	Not revealed
	Bones^1^	- Consume by pregnant women	Believed to be a strong animal
		- Fortify man: grind and mix with *Aframomum melegueta,* water and leaves of *Ageratum cornisoides* and applied on body cuts	Believed to be a strong animal
		- Fortify children: purge by pregnant women and apply on body cuts made on children	Believed to be a strong animal
	Skin^2^	- Making of drums	Resistant to stress
	Meat^4^	- For death, marriage and cultural ceremonies: cook in meals	Traditional inherited practice
African civet *Civettictis civetta*	Anus^1^	- Treats convulsions: inhaled by children because of its pronounced odour	Medicinal
	Nails^4^	- Close two mystical of the four eyes of sorcerers: mix with *Ageratum cornisoides* and perform rituals	Deliverance from evil
	Limbs^1^	Ease walking of children: mix with herbs and purge into children	Medicinal
	Skin^2,3,4^	- Decoration: hang in houses of members of Ekpe society (Figure 8)	Strong and prestigious animal and can be used in replacement of the skin of the leopard
			Denotes hierarchy between the members of Ekpe society
		- Making of drums	Gives the desired sound since it is thick and resistant
		- Used as a carpet by chiefs and elders	Demonstrates hierarchy between members of Ekpe
		- For initiation ceremonies and decoration of the chair of the chief of Ekpe	Replaces the skin of the leopard
	Fur^1^	- Treats convulsions: apply on the eyes of children with palm kernel oil	Medicinal
	Tail^3^	- Decoration	Traditional inherited practice
	Testes^1^	- Treats sexual weakness of men: grind and mix with leaves of «besug-etig»	Medicinal
Preuss’s red colobus *Procolobus preussi*	Skull^1^	- Treats cough/tuberculosis: burn, grind, mix with oil palm and consume	Medicinal
		- Sold to Nigerians	For money
	Bones^1^	- Renders man strong and active: grind and apply on cuts made on the skin	Believed to be a strong animal
	Bones of limbs^1^	- Fortify children: grind and mix with leaves (locally called «Njichondick» or blood leaves). The resulting solution is purged by pregnant women	Believed to be a strong animal
	Limbs and head^1^	- Sold to Nigerians	For money
	Skin^2^	- Making of drums	Resistant
	Fur^1^	- Treats skin burns and dries fresh wounds: plaster on the burned part	Medicinal
		- Treats cough: mix with oil palm, *Ageratum cornisoides* and the coat of palm kernel fruits and consume	Medicinal
Western tree hyrax *Dendrohyrax dorsalis*	Skin^2^	- Making of drums	Light and easily malleable
Drill *Mandrillus leucophaeus*	Skull and limbs^1^	- Sold to Nigerians	For money
	Teeth^3^	- Making necklaces	For prestige
	Bones^1,2^	- For drumming	Traditional inherited practice
		- Treats fractures: tie around fractured hand or leg	Medicinal
	Skin^2^	- Making of drums	Gives a good sound, resistant and thick
Flying squirrel *Funisciurus sp.*;	Fur^1^ (Figure 9)	Treats fire burns and dries fresh wounds: plaster on the burned part and wounds	Medicinal
Tropical forest elephant *Loxodonta cyclotis*	Dung^1^	- Treats stomachache: consume	Medicinal
		- Treats sterility in women: consume	Medicinal
	Hoofs^1^	- Treats elephantiasis: cut into seven parts, and burned then mixed with wood ash. The infected leg is cut using a razor blade and placed on the smoke produced by the fire in which the hoofs are being burned. This is done for seven days	Medicinal
	Tusks^1,3,4^	- Sold to Nigerians	For money
		- Making necklaces and jewelry	For chiefs and elders
		- Decoration	For prestige
		- Protection	Traditional inherited practice
	Bones^1^	- Treats waist pains: grind, mix with the bone marrow and apply on cuts made on the waist with a razor blade	Medicinal
		- Sold to Nigerians	For money
	Skin of the ear^2^	- Making of drums	Solid, durable, resistant and elastic
	Tail fur^3^	- Decorate the cap of chiefs	For prestige
	Meat^4^	- Liberation ceremonies	Not revealed
Western gorilla *Gorilla gorilla*	Skull and limb bones^1^	- Sold to Nigerians	For money
	Right hand bones^1,2^	- Fortify man: grind and mix with palm oil then apply on cuts made on the body with a razor blade	Believed to be a strong animal
		- Fortify babies and children: grind and mix with seeds of *Aframomum melegueta* and used for purging pregnant women	Believed to be a strong animal
		- For drumming	Traditional inherited practice
		- Fortify children: massage of children	Believed that the gorilla is a strong animal
	Skin^2^	- Making of drums	Produces quality sound
	Meat^1^	- Fortify foetus: consume by pregnant women	Believed to be a strong animal
Putty nosed Guenon *Cercopithecus nictitans*	Skin^2^	- Making of drums	Resistant, produces quality sound and it is elastic
Leopard *Panthera pardus*	Teeth^3,4^	- Decoration	Traditional inherited practice
		- Protection: wear as necklaces by chiefs	Traditional inherited practice
	Limbs^1^	- Fortify foetus: grind and mix with cold water and ground roasted plantains. The resulting concoction is purged by pregnant women	Believed to be a strong animal
	Skin^3^	- Decoration of homes of Ekpe members	Signifies prestige and denotes hierarchy between members of Ekpe society
		- Dressing	Hierarchy
		- Making drums played only by Ekpe members	Shows strength of the society and produces quality sound
		- Sold to Nigerians and village chiefs	For money
		- As carpet and for decorating the chair of the chief of Ekpe society	Traditional inherited practice
Marsh mongoose *Atilax paludinosus*	Teeth^1^	- Treats snake bites: used as a blade to wound the bitten part before applying the remedy	Medicinal tool
Mona guenon *Cercopithecus mona*	Skull^1^	- Treats whooping cough: burn, grind and mix with oil palm and consume by children	Medicinal
	Bones^4^	- For charming: mix small pieces with roots of *Mimosa invisa* and put into the pocket. Then call the name of the desired person several times	Traditional inherited practice
African palm civet *Nandinia binotata*	Skin^2^	- Making of drums	Produces quality sound
		- Decoration: hang in the room of a dead member of the Ekpe society in substitution of the skin of a leopard (Figure 10)	Denotes hierarchy between members of Ekpe
	Tail^4^	- Tie on the hands of women (those who have been proposed marriage) during the ‘monenkim’ traditional dance (dance of women) (Figure 11)	Traditional inherited practice
		- As a scarecrow when drying cocoa	For scaring birds and domestic animals
	Fur1	- Treating fire burns: burn, grind and mix with leaves of *Aframomum melegueta* and apply on the burned part	Medicinal
	Head^1^	- Sold to Nigerians	For money
Pangolin *Phataginus tricuspis/Uronamis tetradactyla*	Scales^4^	- Blade	It is sharp
	Skin^2^	- Making of drums	Produces quality sound, not good for consumption
	Meat^4^	- Seduction: very appreciated by women when cooked	It is believed that the fat attracts women
Brushed-tailed porcupine *Atherurus africanus*	Spines^3,4^	- Decorating the caps of members of Ekpe (Figure 12)	Heritage and denotes hierarchy between members of Ekpe society
		- Used as a fork	It is pointed
	Tail^1^	- Sold to Nigerians	For money
	Meat^1,4^	- Gives respect between members in the Ekpe society: prepare and share with other members	Traditional inherited practice
		- Widely used in all ceremonies	Abundant
Red-river hog *Potamochoerus porcus*	Teeth^3^	- For making necklaces: it is traditionally called ‘masanga’ and is worn by chiefs (Figure 13)	Tradition and prestige
			Denotes hierarchy between members of Ekpe
	Skin^1,2^	- Prevents miscarriage: boil and use the resulting concoction for purging pregnant women	Medicinal
		- Making of drums	Solid
	Head^1^	- Sold to Nigerians	For money
Potto *Perodicticus potto*	Skull and hands^1^	- Fortify children and men: scrape and purge	Believed to transmit its strength to children and men
	Skull and limbs^1^	- Sold to Nigerians	For money
	Right hand and limb bone^1^	- Treats hernia: boil in water and mix with the bark of Okan *Cylicodiscus gabonensis* or burn, grind and mix with water and herbs locally called “Tsinabub” (Figure 14). The resulting concoction is purged by pregnant women and children	Medicinal
		- Strengthen children: burn, grind, mix with herbs locally called “Ntemaker” (Figure 15), “Njichondick or blood leaves” or “Osseleayong leaves” (Figure 16) and purge	Believed to be a strong animal
		- Strengthen man: burn, grind, cook with *Aframomum melegueta* and apply on cuts made on the body using a razor blade	Believed to be a strong animal
		- Promotes breast milk abundance and fortifies kids: cook and consume by women who have just given birth	Medicinal
	Skin^2,3^	- Decoration	Shows the importance of the holder
		- Making of drums	Solid and produces desired sounds
	Fur^1^	- Treat burns: plaster on the skin	The wound dries rapidly
Monkeys *Cercopithecus spp.;*	Skull^2^	- Sold to Nigerians	For money
	Limbs, head and young monkeys^2^	- Sold to Nigerians	For money
	Jaws^1^	- Treats toothache and intestinal worms: burn, grind and mix with *Aframomum melegueta* then consume	Medicinal
	Bones^1,2^	- Musical instrument: used by members of the Obhon society	The bone is big
		- Sold to Nigerians	For money
		- Renders men strong: grind and mix with water then purge	Believed to be a strong animal
	Skin^2,4^	- Making of drums	Durable, produces quality sound, light, resistant and elastic
		- Making of baby carriers	Resistant
	Meat^4^	- Cook during death, marriage and traditional ceremonies	Traditional inherited practice

**Note:** ^1^Animals with food, medicinal and sales values.

^2^Ethnomusical animals and parts used as trophy.

^3^Animals used in decoration and jewelry making.

^4^Magico-religious and multipurpose animals.

**Figure 6:**
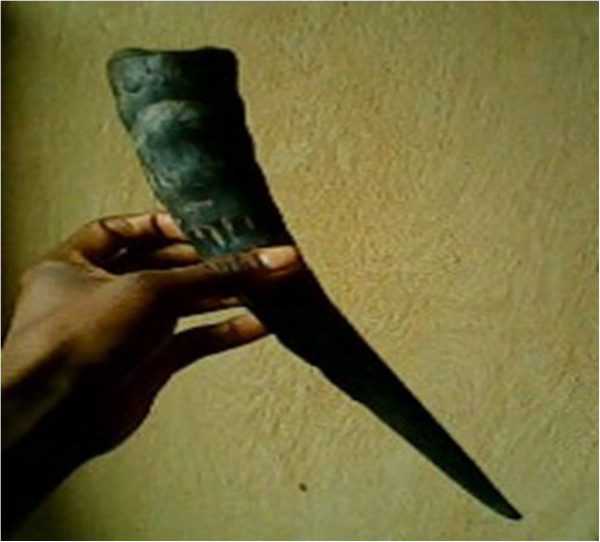
**The horn of an African forest buffalo.**

**Figure 7:**
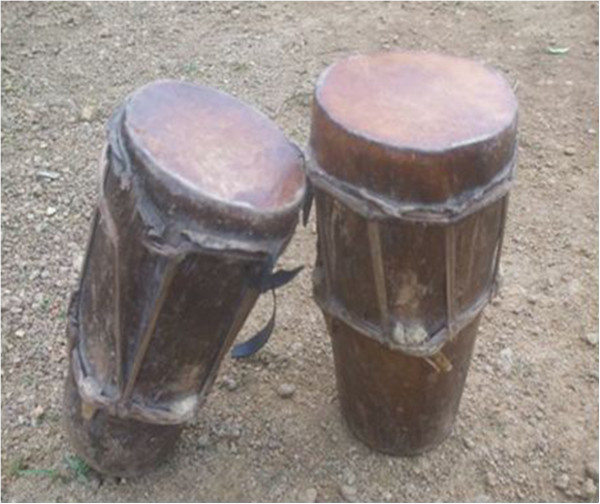
**Skin of the blue duiker.**

**Figure 8:**
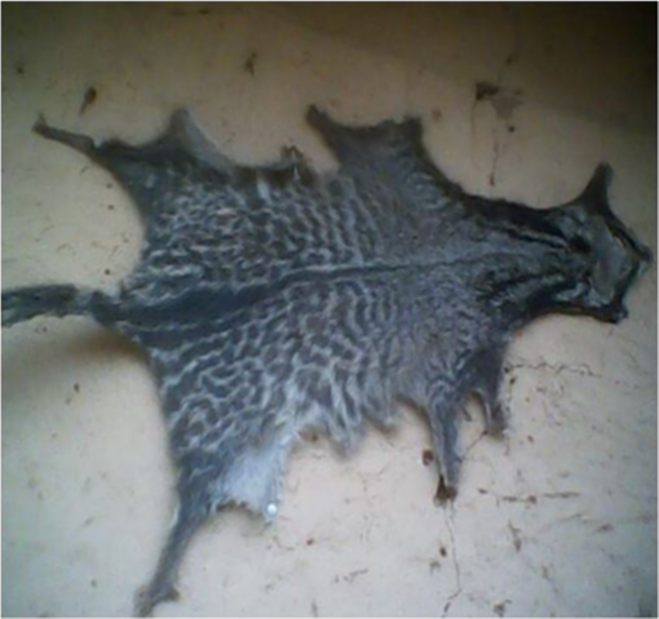
**Skin of an African civet.**

**Figure 9:**
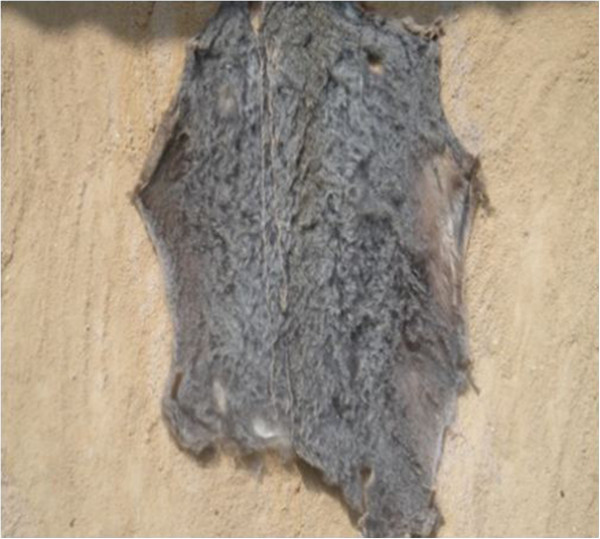
**Skin of the flying squirrel.**

**Figure 10:**
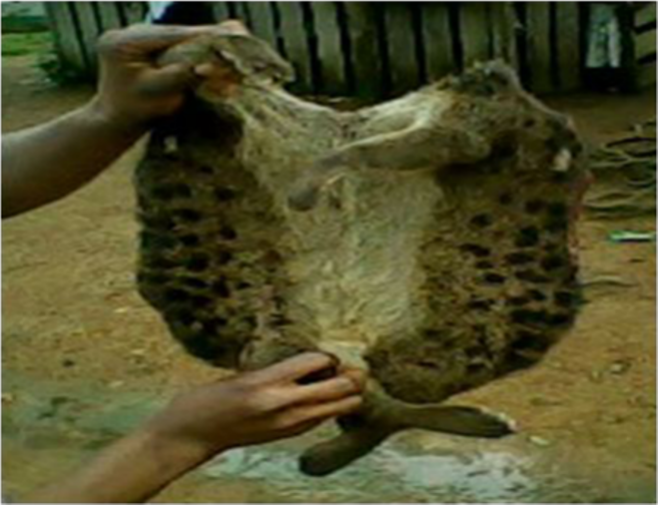
**Skin of an African palm civet.**

**Figure 11:**
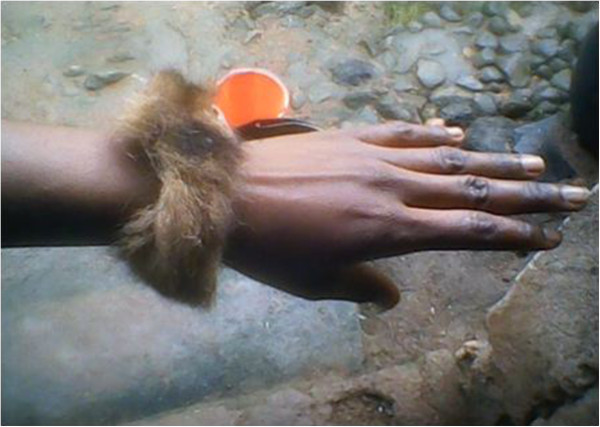
**Tail of an African palm civet.**

**Figure 12:**
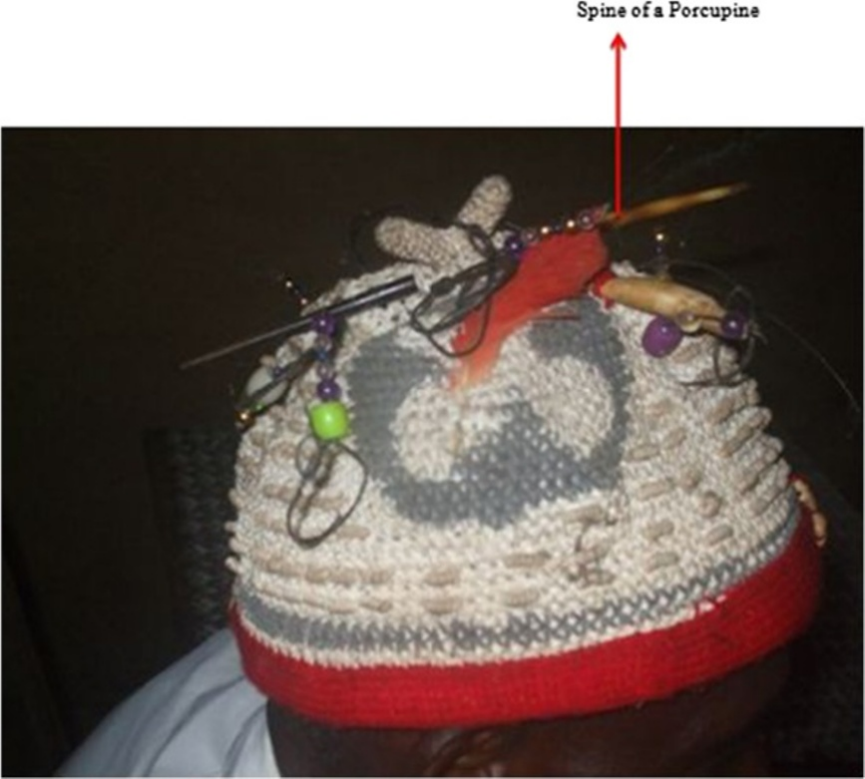
**Spine of the brush tailed porcupine.**

**Figure 13:**
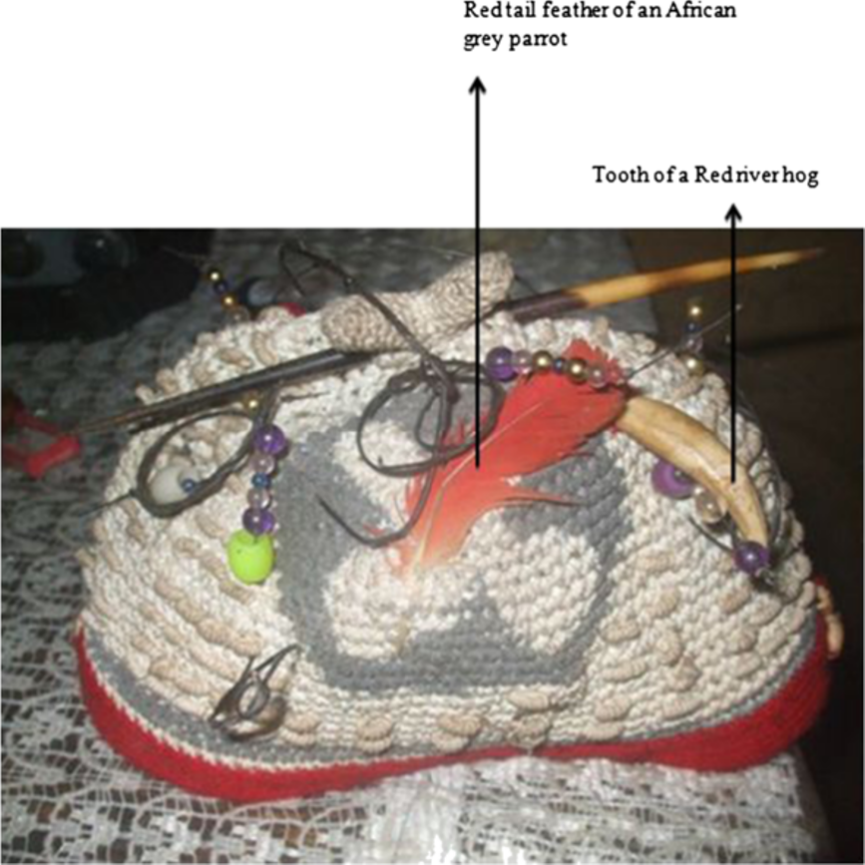
**Tooth of a red river hog and red tail feather of an African grey parrot.** Notes: Both are used in combination to decorate caps of chiefs and elders of sacred societies. Depending on the position of the feather on the cap, it denotes hierarchy between members of sacred societies.

**Figure 14:**
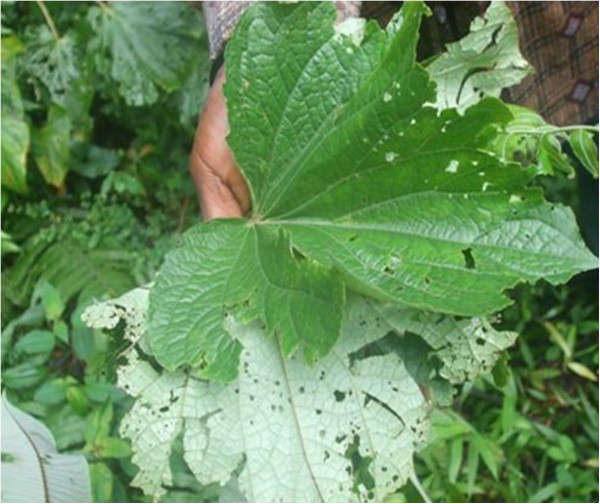
**Tsinabup leaf.** Notes: Unidentified herb important for mixtures of medicine.

**Figure 15:**
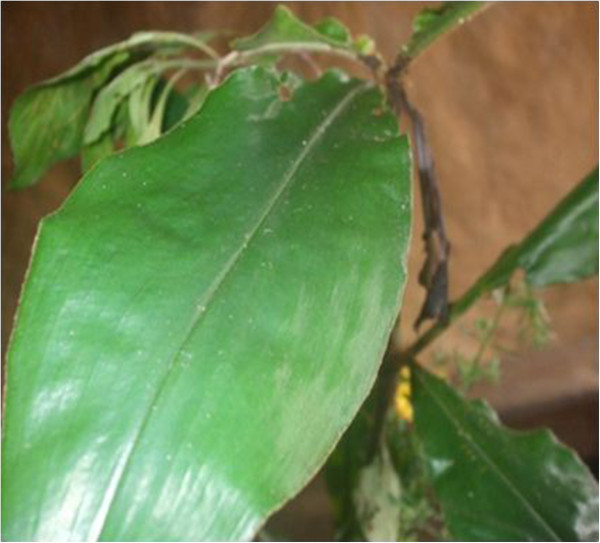
**Ntemaker leaf.** Notes: Unidentified herb important for mixtures of medicines.

**Figure 16:**
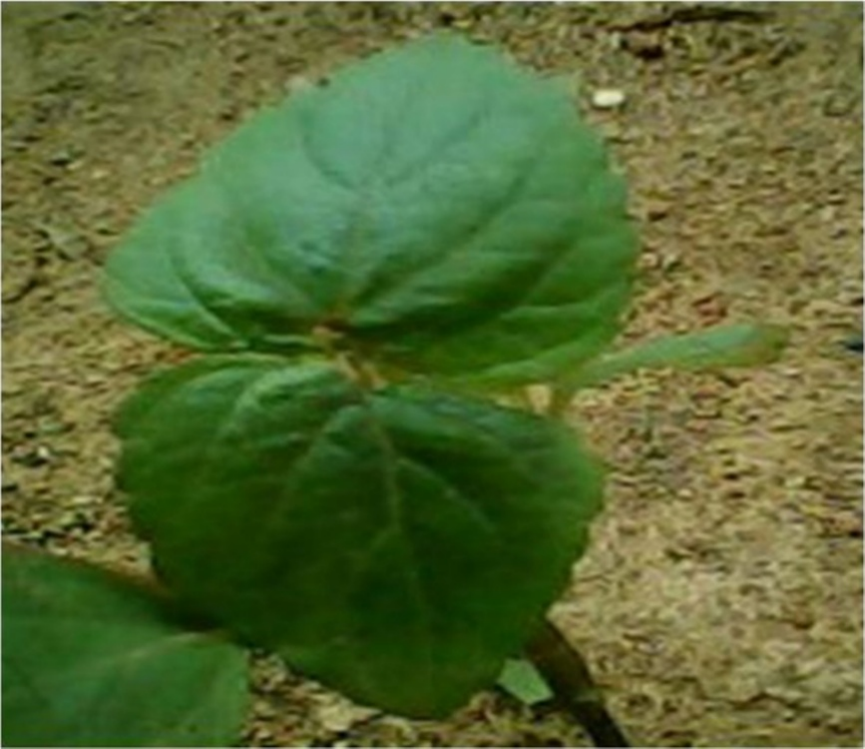
**Osselleayong leaf.** Notes: Unidentified herb important for mixtures of medicines.

**Table 2:** **Birds parts used and reasons**

Animal	Part used	Use and use method	Reason(s)
Crowned eagle *Stephanoaetus coronatus*	Covert feathers^3^	- Decoration	Traditional inherited practice
	Skull and feathers^1^	- Sold to Nigerians	For money
	White feathers^1,3^	- Decoration	Decorate Ekpe sorcerers
		- Wear on caps of members of the Ekpe society and tradi-practitioners	It is a loyal bird and denotes hierarchy between members of the Ekpe
		- Sold to strangers and especially to Nigerians	For money
	Head and limbs^1^	- Sold to Nigerians	For money
Hornbills *Tockus spp.;*	Skull and feathers^1^	- Sold to Nigerians	For money
	Feathers (white and black)^3^	- Decoration	Denotes hierarchy between members of Ekpe
		- Decoration	For Ekpe traditional dance ceremonies
	Head (Figure 17), limbs, feathers and tail^1,4^	- Indicators of witches and wizards: used by tradi-practitioners	Not revealed
		- Sold to Nigerians	For money
	Meat^4^	- For protection: dry, grind and apply on cuts made on the body using razor blade	Traditional inherited practice
Barn owl *Tyto alba*	Head and feathers^1^	- Sold to Nigerians	For money
Black kite *Milvus migrans*	Feathers^3^	- Decorate caps	Denotes hierarchy between members of Ekpe
	Head and limbs^1^	- Sold to Nigerians	For money
	Meat^4^	- Consumed only by elders in traditional meals	Traditional inherited practice
Green sunbird *Anthreptes rectirostris*	Feathers^1^	- Sold to Nigerians	For money
African pygmy kingfisher *Ispidina picta*	Limbs and feathers^1,3^	- Sold to Nigerians	For money
		- Wear on caps of chiefs of Ekpe	Denotes hierarchy between members
African palm Swift *Cypsiurus parvus*	Blood^4^	- Apply on body cuts of women during the “monenkim” dance ceremony	Believed that the women will dance better
Palmnut Vulture *Gypohierax angolensis*	Skull and feathers	- Sold to Nigerians	For money
	Feathers (Figure 18)^1,3,4^	- Decoration of traditional dresses	Denotes hierarchy between Ekpe members
		- For protection: burn, grind and apply on cuts made on the body with a razor blade	Traditional inherited practice
		- Wear on caps	Denotes respect and hierarchy
Black guineafowl *Agelastes niger*	Feathers^3^	- Decoration of caps of members of Ekpe	Denotes hierarchy between Ekpe members
African grey parrot *Psittacus erithacus*	Skull, feathers, tail and limbs^1^	- Sold to Nigerians	For money
	Red tail feathers^1,3,4^ (Figure 13)	- Decorate the masquerades during the Obasinjom traditional dance ceremony	Beautifies the masquerade
		- For protection and decoration: wear on caps of members of Ekpe	Denotes hierarchy between members of Ekpe as it is considered to be a loyal and honored bird. The red feathers represent the bloodshed by ancestors during tribal wars
		- Sold to Nigerians	For money
	Head^1^	- Sold to Nigerians	For money
		- Treats stomachache: grind and mix with the bark of Okan *Cylicodiscus gabonensis* and Ilomba *Pycnanthus angolensis* then purge	Medicinal
	Head and feathers^1^	- Sold to Nigerians	For money
Great blue turaco *Corythaeola cristata*	Feathers^1,3^ (Figure 19)	- Treats whooping cough: burn and mix with medicines and palm kernel oil	Medicinal
		- Decoration	Denotes hierarchy between members of Ekpe
		- Wear by the Obasinjom masquerade	Traditional inherited practice
	Blue feather^3^	- Decoration	Identifies members of sacred societies
	Feathers^1^	- Sold to Nigerians	For money
	Head^1^	- Sold to Nigerians	For money

**Note:** ^1^Animals with food, medicinal and sales values.

^2^Ethnomusical animals and parts used as trophy.

^3^Animals used in decoration and jewelry making.

^4^Magico-religious and multipurpose animals.

**Figure 17:**
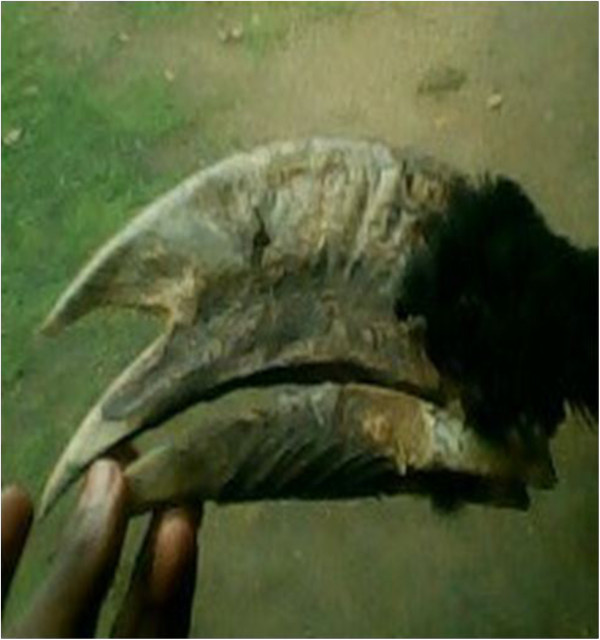
**The head of a hornbill.**

**Figure 18:**
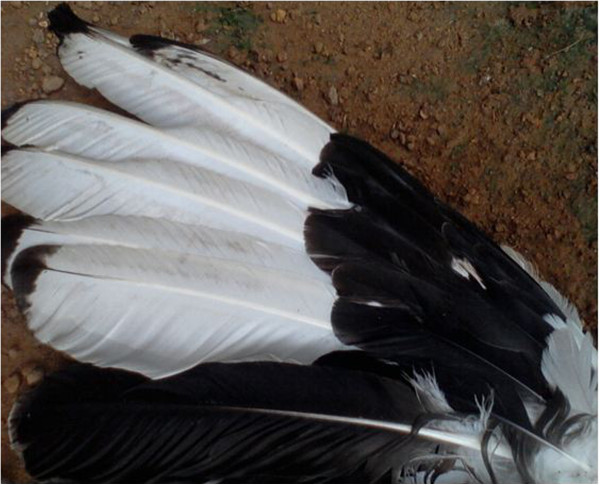
**The feathers of a palmnut vulture.**

**Figure 19:**
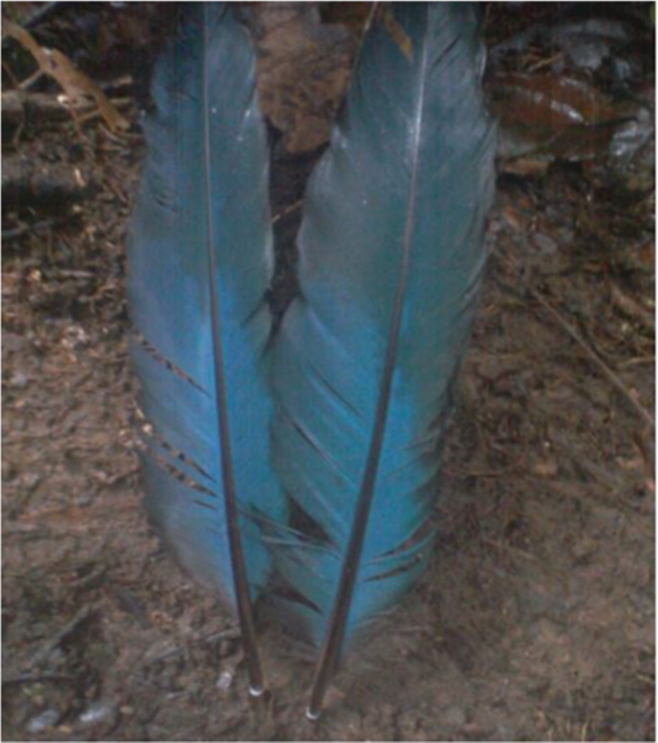
**The feathers of a great blue turaco.**

**Table 3:** **Reptile parts used and reasons**

Animal	Part used	Use and use method	Reason(s)
Nile crocodile *Crocodylus niloticus*	Skull^2^	- Decoration	Traditional inherited practice
	Skin^2^	- Making of drums	Large, durable, elastic and resistant, produce the desired sound (different from other skins) and contains less fat
Egyptian cobra *Naja haje*	Skin^2^	- Making of drums	Strong
	Head^1^	- Sold to Nigerians	For money
Dwarf crocodile *Osteolaemus tetraspis*	Skull and dung^1^	- Sold to Nigerians	For money
	Dung^1^	- Treat skin problems: rub on the skin	Medicinal
	Teeth^3^	- For making necklaces	Prestige
	Skin^2,3^	- Making of drums	Strong and durable
		- Decoration	Indicates a member of Ekpe
African rock python *Python sebae*	Skull^1^	- Sold to Nigerians	For money
	Fat^1^ (Figure 20)	- Treat joint pains, snake bite, fire burns, stomachache, swollen fingers, sprain and skin inflammations: apply on the body part	Medicinal
	Egg^1^	- Treats poison: extract the yolk and apply on cuts made on the body with a razorblade	Medicinal
	Bone and fat^1^	- Treats waist pains: burn and grind the bones, then mix with the fat and apply on cuts made on the waist with a razor blade	Medicinal
	Skin^2,3^	- Making of drums	Durable, produces the desired sound, light, large and elastic
		- Making drums of sacred societies	Denotes strength and power
		- Used as carpets by chiefs	For honor
	Head^1^	- Treats snake bites: grind and mix with your blood and consume	Medicinal
		- Sold to Nigerians	For money
Eastern green mamba *Dendroaspis angusticeps*	Head^1^	- Ingredient for the composition of the remedy against snake bites	Not revealed
Tortoise *Kinixys spp.;*	Shell^1,3,4^ (Figure 21)	- Prevents many diseases: grind and mix with modern medicines and consume	Increases human resistance to diseases
		- To announce messages/sad news in the village: play using either branches of trees, bones of blue duiker, elephant, red river hog, gorilla, drill and chimpanzee	Inherited traditional practice
		- Decoration	Inherited traditional practice
		- Sold to Nigerians	For money
		- Treats eczema: burn, grind and apply on the skin	Medicinal
	Bone^1^	- For strength: burn, grind, mix with oil palm and apply on cuts made on the body with a razor blade	Medicinal
		- Treats fractures: tie on the broken leg	Believed to be a strong animal
	Blood^1^	- Treats eczema: apply on the infected part	Medicinal
	Tortoise^1^	- Sold to tradi-practitioners as it is believed to protects man against sorcerers	For money
Nile monitor *Varanus niloticus*	Skin^2,3^ (Figure 22)	- Making of drums	Durable, resistant, light, elastic and produces good sound
		- Decoration: hang in houses of Ekpe members	Denotes power
	Chest skin^2^	- Making of drums	Solid
Rhinoceros horned viper *Bitis nasicornis*	Teeth^1,4^	- Treats breast pains and induces flow of maternal milk: the breast is pierced several times with the tooth	Inherited traditional practice
		- Treats boyls : pierce the boyl with the tooth	Medicinal
		- Used as a blade	Inherited traditional practice
	Skull^1,4^	- Protect man against sorcerers: grind and mix with oil palm and apply on cuts made on the body with a razor blade	Usually considered as a witchcraft animal
		- Sold to Nigerians	For money
	Skin^2^	- Making of drums	Durable, large, light, elastic and produces quality sound. Also, it does not contain fat
	Head^1^	- Dry and sell to tradi-practitioners and Nigerians	For money

**Note:** ^1^Animals with food, medicinal and sales values.

^2^Ethnomusical animals and parts used as trophy.

^3^Animals used in decoration and jewelry making.

^4^Magico-religious and multipurpose animals.

**Figure 20:**
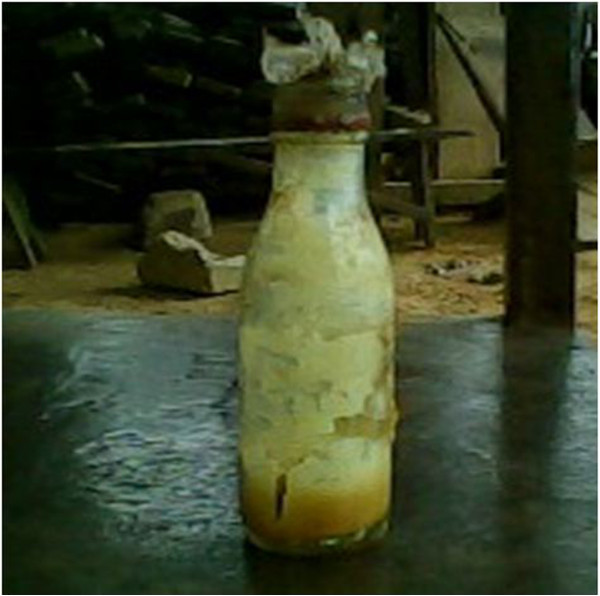
**Fats extracted from the African rock pythons skin.**

**Figure 21:**
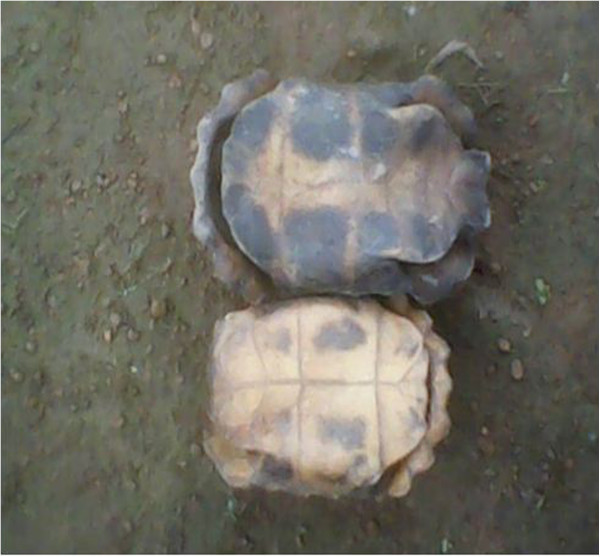
**The shells of tortoises.**

**Figure 22:**
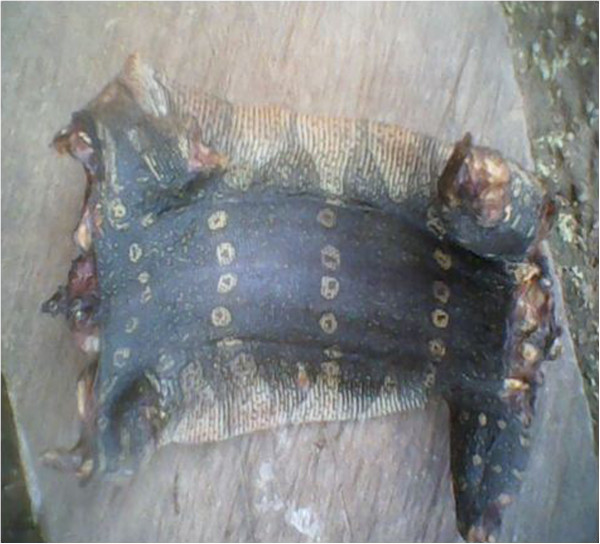
**The skin of a Nile monitor.**

**Table 4:** **Fish parts used and reasons**

Animal	Part used	Use and use method	Reason(s)
Sardine fish *Sardina sp.;*	Young^1^	- Treats cardiovascular illnesses: cook with plenty of oil palm and consume	Medicinal
	Flesh^1^	- Treats cardiovascular illnesses: consume	Medicinal
Electric catfish *Malapterurus electricus*	Flesh^1^	- Treats cardiovascular illnesses: consume	Medicinal
Mud sucker *Labeo coubie*	Flesh^1^	- Treats cardiovascular illnesses: cook and consume	Medicinal
Giant mud fish *Heterobranchus sp.*;	Flesh^1^	- Treats cardiovascular illnesses: cook and consume	Medicinal

**Note:** ^1^Animals with food, medicinal and sales values.

^2^Ethnomusical animals and parts used as trophy.

^3^Animals used in decoration and jewelry making.

^4^Magico-religious and multipurpose animals.

**Table 5:** **Invertebrates parts used and reasons**

Animal	Part used	Use and use method	Reason(s)
Snails *Achatina sp.*;	Shell^1,2,3^	- Formerly used a drinking cup	Drinking cups did not exist before
		- Musical instrument	Produces a particular unique sound
		- Decoration	Traditional inherited practice
		- Treats waist problems: grind and mix with leaves of *Ageratum cornisoides* and apply on cuts made on the body with a razor blade	Medicinal
		- Key holders	Inherited traditional practice
	Sticky/slippery liquid from the body	- Eases birth (reduces labour pains): mix with water and purge by pregnant women	Believed to make the foetus slide out easily
Crabs *Emerita sp.;/Blepharipoda sp*.;	Carapace^1^	- Treats eczema: burn and mix the ash with Tsinabup leaves and apply on the skin	Medicinal
	Chest^1^	- Prevents children from watering the bed at night: consume by children	Medicinal

**Note:** ^1^Animals with food, medicinal and sales values.

^2^Ethnomusical animals and parts used as trophy.

^3^Animals used in decoration and jewelry making.

^4^Magico-religious and multipurpose animals.

Some of the uses concerning mammals and reptiles correspond to those described by [49] at the periphery of Rumpi hills forest reserve, and by [18] in the Korup area for mammals only. Some of the uses concerning some of the birds species described in this paper were also identified in [18, 50]. Some of the uses concerning fish and invertebrates correspond to those described in [51].

Adapted from [22], we classified the above uses of wildlife in the study area in four categories namely:

#### Animals with food, medicinal and sales values

This category represented 41.2% of all uses of wildlife in the study area. This group contains animals like Ogilby’s duiker, blue duiker, flying squirrel, potto, chimpanzees (Table 1), African grey parrot, hornbills (Table 2), African rock python, rhinoceros horned viper (Table 3) and all fish (Table 4). According to [19–21]; [48–51], the overlapping of food and medicinal uses is a common finding from India and other parts of the world. Such animals with dual role are important for healing and providing nourishing food items to boost up the immune system [22]. In the present paper they were also considered as a source of income. It should also be noted that the culture regulates the use of species especially those of nutritional and medicinal importance [19–22].

#### Ethnomusical animals and parts used as trophy

This category represented 29.2% of all uses of wildlife in the study area. The skin is the main part used for making drums. Animals whose skins are elastic, resistant, durable and non fatty are preferred. Species like blue duiker, Ogilby’s duiker, bay duiker (Table 1), African rock python, dwarf crocodiles and Nile monitor (Table 3) are cherished (see also [18]).

#### Animals used in decoration and jewelry making

This category represented 21.9% of all uses of wildlife in the study area. The main parts used are the teeth, feathers of birds and skins. Parts of African civet, leopard, Red river hog (Table 1), African grey parrot, crowned eagle, palmnut vulture (Table 2), African rock python and tortoise (Table 3) are preferred.

#### Magico-religious and multipurpose animals

This category represented 7.8% of all uses of wildlife by both communities. These animals are generally used to communicate with ancestors. Species like Ogilby’s duiker, leopard, African civet (Table 1), and crowned eagles (Table 2) are preferred. This category is also represented by animals whose parts serve in various ways such as the buffalo and elephant. Like the Tamang community in Central Nepal, the populations of the study area maintain not only material level relationships with animals but also spiritual relationships [22]. This has been reported in different parts of the world [52, 53].

### Cultural taboos

With respect to wildlife use, four types of taboos were identified. These are habitat, species specific, method and segment taboos (see also [54–56]). Among taboo species we found totems that differ according to their origin. On one hand, we found totems which are acquired at birth known as inborn or generation totems, e.g. red river hog, drill, forest elephant and chimpanzee. On the other hand, we found voluntary acquired totems or personal totems such as red-capped mangabey, African rock python and owls. These personal totems are purchased in spiritual markets with the aid of native doctors. These totems play great roles in the conservation of certain species because it is believed that when a totem is killed, a person will die in the village or neighboring village. As concerns habitat taboos, sacred forests exist in the study area. According to [13] they are crucial conservation sites characterised by high biodiversity. They varied between one and four sites per village. The four locally identified sacred forests are Ekpe, Mawooh, Obhon and Amgbu sacred forests. Habitat taboo was the most common in the study area and seems to be the most known and respected form of taboo in Africa [55]. The restriction to use gamaline and toxic products for fishing by the village traditional councils was the main method taboo. Segment taboos are manifested by the restriction of women and children from consuming certain animals such as Red river hog (according to 15.6% of HRs), Snakes (according to 8.5% of HRs) and most primates. It is however important to note that threats to wildlife is real. An evaluation of the perceptions of HRs on the use level of wildlife showed that, wildlife use for cultural and traditional purposes is disappearing progressively (according to 96.7% of HRs). This trend was mainly because of the scarcity of wildlife (65.3% of HRs) and the loss of culture among the youths (12.5% of HRs).

## Conclusion

Literature on efficacy of indigenous knowledge offers huge hopes to conservation success [57–60]. Like other indigenous populations, the Obang and Ngunnchang populations possess rich ethnozoological knowledge. They have accumulated knowledge on the uses of several species of wildlife which were classified into animals with “food, medicinal and sales values”, “ethnomusical animals and parts used as trophy”, animals used in “decoration and jewelry making” and “magico-religious and multipurpose animals” based on the method of use and the reason attributed by HRs (see also [22]). Body parts such as the skin are used in making musical instruments such as drums. Flesh cooked with plantains and palm oil is used for liberation ceremonies and communication with ancestors. Bones are used for strengthening; feathers are used to denote hierarchy between members of sacred societies and many others are used for medicines. Plants like *Ageratum cornisoides, Aframomum melegueta* and *Elaeis guineensis* happen to be very important for mixtures of medicines and thus play important medicinal roles (see also [50]). Both communities believe in species specific, habitat, method and segment taboos which, if respected duly, are great conservation measures. They are traditional practices known to have promising potential for enhancing sustainable resource use (see also [61]). To evaluate the level of respect of the culture in other to ascertain the role played by traditional knowledge and taboos in conservation, it is imperative to know the level of loss of the culture (acculturation) as the conservation of not only biodiversity but also cultural diversity is necessary for development [22]. Strategies to fight against threats to wildlife should thus be defined taking also into consideration traditional knowledge and taboos. High priority should be given to reconciling conservation of species with high values for local communities and human needs.
